# Twelve-Month Longitudinal Serology in SARS-CoV-2 Naïve and Experienced Vaccine Recipients and Unvaccinated COVID-19-Infected Individuals

**DOI:** 10.3390/vaccines10050813

**Published:** 2022-05-20

**Authors:** Zion Congrave-Wilson, Wesley A. Cheng, Yesun Lee, Stephanie Perez, Lauren Turner, Carolyn Jennifer Marentes Ruiz, Shirley Mendieta, Adam Skura, Jaycee Jumarang, Jennifer Del Valle, John Kubale, Emma Kaitlynn Allen, Paul G. Thomas, Aubree Gordon, Pia S. Pannaraj

**Affiliations:** 1 Division of Infectious Diseases, Children’s Hospital Los Angeles, Los Angeles, CA 90027, USA; zcongravewilson@chla.usc.edu (Z.C.-W.); wcheng@chla.usc.edu (W.A.C.); yelee@chla.usc.edu (Y.L.); stephanieperez@gmail.com (S.P.); lturner@chla.usc.edu (L.T.); cmarentesruiz@chla.usc.edu (C.J.M.R.); smendieta@chla.usc.edu (S.M.); askura@chla.usc.edu (A.S.); jjumarang@chla.usc.edu (J.J.); jdelvalle@chla.usc.edu (J.D.V.); 2 Department of Epidemiology, School of Public Health, University of Michigan, Ann Arbor, MI 48109, USA; jkubale@umich.edu (J.K.); gordonal@umich.edu (A.G.); 3 Department of Immunology, St. Jude Children’s Research Hospital, Memphis, TN 38105, USA; emma.allen@stjude.org (E.K.A.); paul.thomas@stjude.org (P.G.T.); 4 Department of Pediatrics and Molecular Microbiology and Immunology, Keck School of Medicine, University of Southern California, Los Angeles, CA 90033, USA

**Keywords:** SARS-CoV-2, COVID-19, vaccination, IgG, IgA, neutralizing antibodies, 12 months, serum, prior infection, longitudinal

## Abstract

Longitudinal data comparing SARS-CoV-2 serology in individuals following infection and vaccination over 12 months are limited. This study compared the magnitude, decay, and variability in serum IgG, IgA, and neutralizing activity induced by natural infection (*n* = 218) or mRNA vaccination in SARS-CoV-2 naïve (*n* = 143) or experienced (*n* = 122) individuals over time using enzyme-linked immunosorbent assays and an in vitro virus neutralization assay. Serological responses were found to be highly variable after natural infection compared with vaccination but durable through 12 months. Antibody levels in vaccinated, SARS-CoV-2 naïve individuals peaked by 1 month then declined through 9 months, culminating in non-detectable SARS-CoV-2-specific serum IgA. Individuals with both infection and vaccination showed SARS-CoV-2-specific IgG and IgA levels that were more robust and slower to decline than the other groups; neutralizing activity remained highest in this group at 9 months past vaccination. These data reinforce the benefit of vaccination after SARS-CoV-2 recovery.

## 1. Introduction

Severe acute respiratory syndrome coronavirus-2 (SARS-CoV-2) infection and vaccination confer protection against re-infection and severe COVID-19 and may also reduce viral transmission [1,2]. Serum spike-specific IgG and neutralizing antibodies against SARS-CoV-2 have been reported as correlates of protection [3,4]. SARS-CoV-2-specific IgG persists but wanes following SARS-CoV-2 infection and the primary vaccine series prior to an additional dose [5,6,7,8,9,10,11,12]. Past studies have compared spike-specific IgG levels post-vaccination between individuals who experienced a SARS-CoV-2 infection before vaccination and naïve individuals, mainly at early time points [12,13,14,15,16]. However, few studies have compared SARS-CoV-2-specific IgG longitudinally in individuals following infection, vaccination, and both infection and vaccination [16,17,18].

The role of each antibody isotype in virus neutralization and prevention of illness is still unclear. Researchers have found that the serum antibody response during the acute phase of illness and early in convalescence is predominantly of the IgA isotype [19,20]. Secretory IgA (sIgA) plays a crucial role in immunity at mucosal barriers [21] and has been reported as protective in other respiratory viral disease settings [22,23]. This antibody isotype may also be involved in preventing SARS-CoV-2 entry into host cells and slowing replication at the first point of contact [24]. SARS-CoV-2-specific IgA production in the serum and at mucosal linings is elicited by COVID-19 vaccination despite the current intramuscular administration route [25,26]. Studies comparing the longevity of the IgA response in vaccinated individuals compared with those who experienced natural infection are limited [27,28].

Here we conducted longitudinal serological testing in a prospective cohort of three groups: unvaccinated individuals following SARS-CoV-2 infection (infection-only), vaccinated individuals without prior SARS-CoV-2 infection (vaccination-only), and vaccinated individuals with prior infection (infection + vaccination). This study sought to evaluate serum IgG and IgA responses up to 12 months post-infection and 9 months post-COVID-19-vaccination, compare differences in antibody magnitude between individuals based on SARS-CoV-2 infection status, and characterize serum SARS-CoV-2 neutralization potential over time.

## 2. Methods

### 2.1. Study Design and Participants

Participants were enrolled into one of two COVID-19 cohorts in Los Angeles, CA from Children’s Hospital Los Angeles (CHLA) and nearby community SARS-CoV-2 testing sites using a convenience recruitment strategy. Individuals were enrolled in the Household Exposure and Respiratory Virus Transmission and Immunity Study (HEARTS) if they were exposed to a household member who tested positive for SARS-CoV-2 by RT-PCR within 2 weeks [29]. Only those who tested positive for SARS-CoV-2 were included in the analyses described here. Individuals were enrolled in the SARS-CoV-2 Antibody and Immunity from Natural Infection and The vaccine (SAINT) study if they were planning to receive a COVID-19 vaccine within 1 month. There were no exclusion criteria if the individual met the inclusion criteria.

Demographic information, co-morbidities, COVID-19 exposure, and SARS-CoV-2 testing/infection history were obtained via questionnaire at enrollment for both studies. Both studies were approved by the institutional review board of CHLA and written informed consent was obtained from all participants or their parents/legal guardian. Assent was obtained from all children ≥7 years old.

### 2.2. Specimen Collection and Processing

All specimens were collected from participants in the HEARTS and SAINT studies by clinical research staff between June 2020 and November 2021. For HEARTS enrollees, nasopharyngeal swab (NPS) samples were collected at enrollment and every 4–7 days if RT-PCR positive for SARS-CoV-2 until 2 negative tests. Blood samples were collected at enrollment and then at convalescent visits 1 month after resolution of NPS RT-PCR positivity and every 3 months thereafter. For SAINT enrollees, blood was collected before COVID-19 vaccination, 7–14 days after the second vaccine dose, and then every 3 months after the first dose. Blood and NPS samples were transported to the laboratory within 2 h after collection. Serum was extracted from coagulated blood via centrifugation and stored overnight at −20 °C for next-day serology testing. NPS specimens were collected in 3 mL universal transport media tubes and stored as 200 µL aliquots at 4 °C for next-day RNA extraction and RT-PCR.

### 2.3. SARS-CoV-2 Serology

Serum anti-SARS-CoV-2 receptor-binding domain (RBD) IgG and IgA and spike IgG antibody was measured using a previously described ELISA [30]. The threshold for positivity was set at the optical density at 490 nm (OD_490_) value of 0.200 for RBD-specific IgG and 0.104 for RBD-specific IgA, calculated from the mean OD_490_ plus 3 standard deviations (SD) from 20 archived serum samples collected before the initial SARS-CoV-2 outbreak and from the published protocol [30]. For anti-RBD IgG positive samples, IgG against the spike protein was used to quantify the response. As some responses could not be quantified over a maximum OD_490_ value, spike-specific IgG was reported as the area under the curve (AUC). AUC values were calculated using the spike-specific IgG OD_490_ values from 5 serial dilutions (1:100–8100) in all samples which were positive for RBD-specific IgG. Serum testing under the positive threshold for RBD-specific IgG was given an arbitrary AUC of 1.

### 2.4. SARS-CoV-2 Virus Neutralization Assay

Serum antibody neutralization activity against SARS-CoV-2 was measured using a commercially available surrogate virus neutralization test kit (GenScript, Piscataway, NJ, USA), which showed high correlation with the 90% plaque reduction neutralization test (Pearson R = 0.84, *p* < 0.01) [31]. Briefly, 60 μL of serum (diluted 1:10 in sample dilution buffer) was mixed with an equal volume of horseradish peroxidase conjugated to SARS-CoV-2 RBD protein and incubated for 30 min at 37 °C. Then, 100 μL of each mixture was added to each well on an angiotensin-converting enzyme 2 coated microtiter plate. Remaining steps were performed according to the manufacturer’s protocol. The assay was performed in duplicate. The percent inhibition of each sample was calculated as Inhibition (%) = (1 − Sample OD_450_ value/Negative Control OD_450_ value × 100). The mean plus 3 SD percent inhibition from 20 serum samples collected from healthy individuals between 2017 and 2019 was 3.5% inhibition; however, we used a more conservative positive threshold of 30% inhibition based on the manufacturer’s protocol.

### 2.5. SARS-CoV-2 Reverse Transcription Real Time Polymerase Chain Reaction (RT-PCR)

RT-PCR for the SARS-CoV-2 N1 and N2 genes was performed in accordance with the guidelines from the Centers for Disease Control and Prevention and as previously published by our group [32]. A cycle threshold (Ct) value of <40 for the N1 and N2 genes was considered positive for SARS-CoV-2 infection. Positive PCR results were validated using a cutoff Ribonuclease P internal control Ct of <32.

### 2.6. Definitions

For vaccinated participants, time since vaccination was determined from the date of the first vaccine dose. For naturally infected participants, COVID-19 onset date was defined as the earlier date between first RT-PCR positivity and COVID-19-associated symptom onset. If a seropositive participant was asymptomatic, their COVID-19 onset date was defined as the average onset date in the household. We classified the blood collection time points after vaccination or COVID-19 onset as follows: 2 weeks to less than 60 days = “1 month”, 60 to 149 days = “3 months”, 150 to 239 days = “6 months”, 240 to 329 days = “9 months”, and greater than or equal to 330 days = “≥12 months”.

### 2.7. Statistics

Nonparametric continuous variables were analyzed using the Kruskal−Wallis test followed by Dunn’s multiple comparisons test with the Benjamini−Hochberg correction if the analysis included 3 or more groups. Paired time points were analyzed using the Wilcoxon signed-rank test. Correlations were computed using the nonparametric Spearman correlation coefficient. Chi-squared or Fisher’s exact test were used to compare proportions. The coefficient of quartile variation (CQV) was calculated as the interquartile range divided by the sum of the 1st and 3rd quartiles and Bonett’s method was used for confidence intervals.

Antibody decay was modeled for participants using a generalized additive mixed model. Separate smoothing functions for days since infection/vaccination were fit to the infection-only, vaccination-only, and infection + vaccination groups along with fixed effects for age and sex. A random intercept was included to account for repeated measures within individuals. Statistical analyses were performed using R Studio v4.0.3 (RStudio, Boston, MA, USA) and GraphPad Prism version 9 (GraphPad Software, Inc., San Diego, CA, USA). All tests were 2-tailed with *p* < 0.05 considered significant.

## 3. Results

### 3.1. Participant Characteristics

Samples were collected between June 2020 and November 2021 from the HEARTS SARS-CoV-2 exposed cohort; 525 participants had SARS-CoV-2 infection confirmed by RT-PCR (443 (84.4%)) or paired acute and convalescent serology (82 (15.6%)). We excluded 160 infected children <12 years old because COVID-19 vaccines were not available for this age group at the time of sample collection. Only samples collected at least 2 weeks after COVID-19 onset and prior to COVID-19 vaccination or suspected re-infection were included in the natural infection longitudinal analysis.

From the SAINT vaccination cohort, 208 participants had serum samples collected before and at least 1 month after vaccination between December 2020 and November 2021. Because only eight (2.9%) vaccinated participants received Ad26.COV2.S (Janssen/Johnson & Johnson), we focused the current analysis on mRNA vaccine recipients. Two participants were excluded due to contracting a SARS-CoV-2 infection between COVID-19 vaccine doses; one participant was excluded for not completing the 2-dose primary series. In the SAINT cohort, 62 (30.2%) had evidence of SARS-CoV-2 infection prior to vaccination by positive RBD-specific IgG. An additional 60 participants from the HEARTS cohort had samples collected at least 1 month after COVID-19 vaccination following natural infection and were added to the infection + vaccination group. Only samples collected prior to a third vaccine dose were included in the analysis. In total, 944 serum samples were collected from 423 individuals (Table S1), including 218 participants in the infection-only group, 143 participants in the vaccination-only group, and 122 participants in the infection + vaccination group.

**Figure 1:**
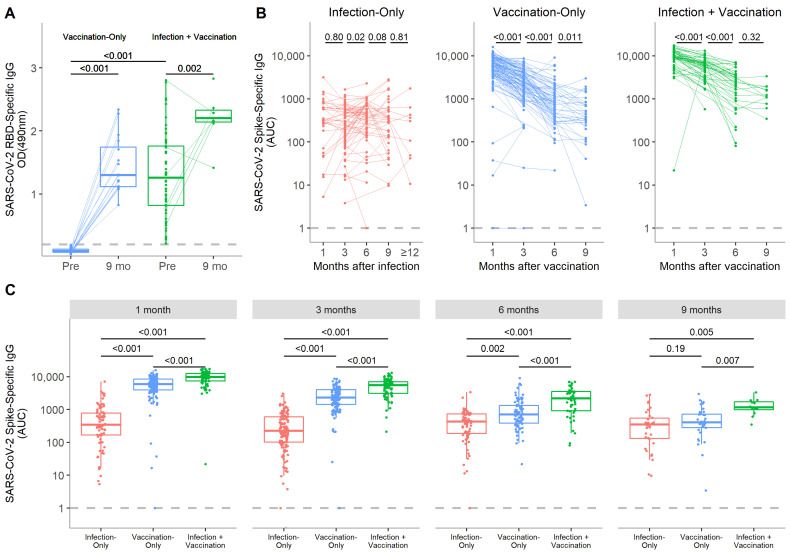
SARS-CoV-2-specific IgG responses over time in the serum following SARS-CoV-2 infection, COVID-19 mRNA vaccination, or both. (**A**) Pre-vaccination levels of RBD-specific IgG in participants in the vaccination-only and infection + vaccination groups are compared with 9 month post-vaccination paired samples. The threshold for positivity was set at 0.200 OD_490_ based on levels observed in pre-pandemic samples. (**B**) Longitudinal analyses of spike-specific IgG over time from paired serum samples are displayed for the infection-only, vaccination-only, and infection + vaccination participants. (**C**) The magnitude of the IgG response at each time point is compared among the infection-only, vaccination-only, and infection + vaccination groups. Spike-specific IgG is reported as area under the curve (AUC) with the box as the median and interquartile range and the whiskers as the minimum and maximum values, excluding outliers. *p*-values were calculated by Wilcoxon signed-rank tests if between paired time points or the Kruskal−Wallis test followed by Dunn’s multiple comparisons test if between multiple groups.

Demographic and clinical characteristics for each group are reported in Table 1. The mean age was 32.5 years (range 12.0 to 84.5), and 38.5% (163/423) of participants were male. A higher proportion of participants reported Hispanic/Latinx ethnicity in the natural infection (92.2%) compared with the vaccinated cohort (61.1%) (*p* < 0.001), reflecting SARS-CoV-2 infection and vaccination trends in California at the time of sample collection [33,34]. Of mRNA-vaccinated participants, 224 (84.5%) received BNT162b2 (Pfizer-BioNTech) and 41 (15.5%) received mRNA-1273 (Moderna). The mean duration between the first and second dose was 22.1 days (SD: 2.9 days) for BNT162b2 recipients and 30.7 days (SD: 10.1 days) for mRNA-1273 recipients. The time between SARS-CoV-2 infection and vaccination for infection + vaccination participants is shown in Table S2. Pre-vaccination anti-RBD IgG levels for the vaccinated cohort are presented in Figure 1A.

### 3.2. SARS-CoV-2-Specific IgG Responses Differ by Experience with Infection, Vaccination, or Both

Nearly all participants in the infection-only group were positive for RBD-specific IgG at all time points through 12 months following COVID-19 onset, except for two individuals who tested negative at 1 and 3 months, respectively, and another who declined below the threshold at 6 months (Table 2). Only one vaccination-only participant, who was on immunosuppressive medication, did not mount a detectable serum IgG response at 1 and 3 months post-vaccination before receiving a third vaccine dose. All individuals tested at 9 months and later in all three groups were above the threshold for positivity.

Infection induced a highly variable IgG response; variation was 1.78 times higher than the vaccination-only group and 2.46 times higher than the infection + vaccination group at 1 month post-COVID-19 onset or vaccination, respectively (Figure S1). This trend was maintained out to 3 months, but by 6 months variation was indistinguishable between groups.

In the infection-only group, no significant difference was seen between spike-specific IgG levels in 1 and 12 month samples (median 343 vs. 326 AUC, respectively, *p* = 0.35), suggesting durable anti-spike IgG to at least a year following infection (Figure 1B). There was an unexpected small increase in IgG from 3 to 6 months following infection (254 vs. 427 AUC, respectively, *p* = 0.02) when looking at paired analyses. In the vaccination-only group, spike-specific IgG levels were highest at 1 month, but decreased out to 9 months post-vaccination (Figure 1B). Spike-specific IgG levels in the infection + vaccination group followed a similar trend and declined out to 6 months (Figure 1B). However, the rate of IgG decay appeared to slow thereafter as the 6 and 9 month time points were not significantly different (*p* = 0.32). Anti-RBD IgG levels at 9 months remained significantly higher than the pre-vaccination baseline for both the vaccination-only and infection + vaccination groups (*p* < 0.001 and *p* = 0.002, respectively) (Figure 1A).

**Figure 2:**
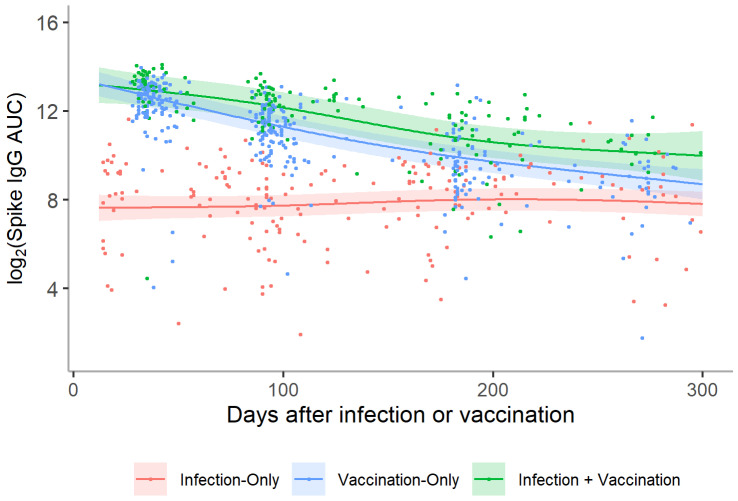
Log_2_ anti-spike IgG titers over time for participants in the infection-only, vaccination-only, and infection + vaccination groups. A distinct smoothing function was fit for paired samples from the infection-only (*n* = 201), vaccination-only (*n* = 346), and infection + vaccination (*n* = 162) groups using a generalized additive model (GAM). The figure represents the mean trajectory for each group marginalized over age and sex. A random intercept was also included to account for repeated measures within individuals. The two vaccine groups displayed higher titers soon after infection/vaccination compared with the infection-only group, gradually decreasing until beginning to approach the titer levels of the infection-only group approximately 300 days post-infection/vaccination.

When modeled over time, higher titers in the vaccinated cohort in the month immediately following vaccination were retained to at least 300 days at which point both the vaccination-only and infection + vaccination groups began to approach the relatively static spike-specific IgG levels of the infection-only group (Figure 2). On average, vaccination-only and infection + vaccination participants displayed titers that were 6.5 (95% CI: 4.8–8.9) and 14.9 (95% CI: 10.5–21.1) times that of infection-only participants, respectively (Table S3). Longitudinally, infection + vaccination participants had the highest IgG levels at all time points out to 9 months (Figure 1C).

### 3.3. SARS-CoV-2-Specific IgA Is Retained through 9 Months in Infection + Vaccination Individuals but Not in Vaccination-Only Participants

Percentages of individuals positive for anti-RBD IgA at all time points are presented in Table 2. The same three infection-only individuals who were negative for IgG were also negative for IgA at the same time points; all other participants were positive. An IgA response was detectable in 95.0% (132/139) of vaccination-only participants by 1 month post-vaccination but decreased to 37.1% (13/35) by 9 months. All infection + vaccination participants were positive for anti-RBD IgA by 1 month post-vaccination and that percentage remained high through 9 months (91.7% (11/12)).

Variation in the IgA response was greatest in the infection-only group at 1 month post-vaccination (Figure S1). However, there was no discernable trend in variation between groups after 1 month, signifying that the IgA response was more predictable in vaccinated compared with SARS-CoV-2-recovered individuals only at early time points.

**Figure 3:**
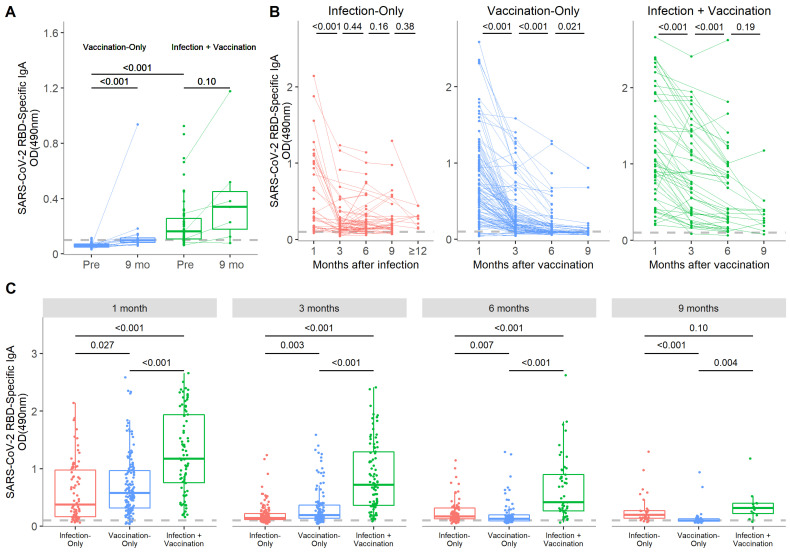
Serum levels of SARS-CoV-2-specific IgA over time after SARS-CoV-2 infection and COVID-19 mRNA vaccination, with or without prior immunity. (**A**) Levels of RBD-specific IgA pre-vaccination are compared with the paired 9 month samples in the vaccination-only and infection + vaccination groups. The threshold for RBD-specific IgA positivity was set at 0.104 OD_490_ based on levels in pre-pandemic samples. (**B**) Analyses of the change in anti-RBD IgA levels over time are presented for paired samples from the infection-only, vaccination-only, and infection + vaccination groups. (**C**) At each time point, the RBD-specific IgA response was compared among samples from the infection-only, vaccination-only, and infection + vaccination groups. Boxes represent the median and interquartile range and whiskers represent the minimum and maximum values. *p*-values were calculated by Wilcoxon signed-rank tests if between paired time points or the Kruskal−Wallis test followed by Dunn’s multiple comparisons test if between multiple groups.

**Figure 4:**
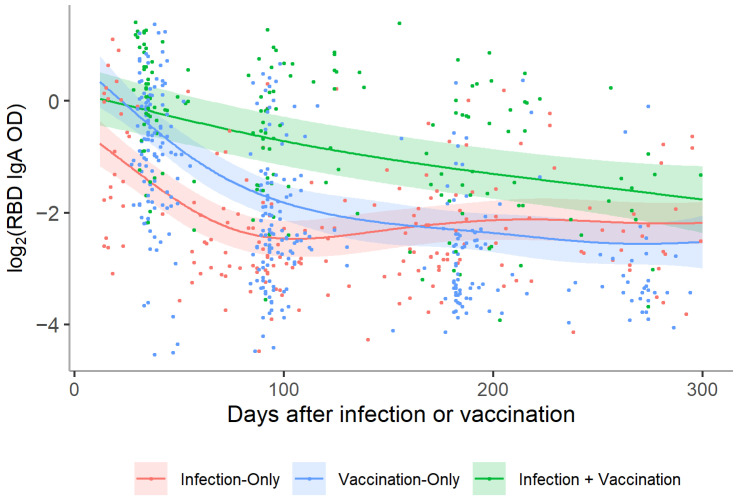
Log_2_ anti-RBD IgA titers over time for participants in the infection-only, vaccination-only, and infection + vaccination groups. A distinct smoothing function was fit for samples from the infection-only (*n* = 201), vaccination-only (*n* = 346), and infection + vaccination (*n* = 162) groups using a GAM. The figure represents the mean trajectory for each group marginalized over age and sex. A random intercept was also included to account for repeated measures within individuals. The two vaccine groups displayed higher titers soon after infection/vaccination compared with the infection-only group; however, the vaccination-only group displayed a similar trajectory to the infection-only group: a more rapid decline in the first 100 days and becoming more stable thereafter. Conversely, the infection + vaccination group displayed a more gradual decline before approaching the levels of the other two groups approximately 300 days post-infection/vaccination.

Serum anti-RBD IgA positively correlated with IgG levels (ρ = 0.49, *p* < 0.001) (Figure S2). The SARS-CoV-2-specific IgA decay trajectory over time differed between groups. In the infection-only group, IgA decreased from 1 to 3 months post-COVID-19 onset (*p* < 0.001) but then did not significantly change out to 12 months, suggesting stabilization of the IgA response (Figure 3B and Figure 4). In infection + vaccination participants, RBD-specific IgA decreased to 6 months (both *p* < 0.001) and then the decay rate seemed to slow out to 9 months post-vaccination (*p* = 0.19). Although most vaccination-only participants dropped below the threshold for IgA positivity by 9 months, levels were still significantly higher than pre-vaccination, even when the outlier was removed (both *p* < 0.001) (Figure 3A).

When modeled over time, both the infection-only and vaccination-only groups experienced a period of more rapid decline in IgA levels before slowing approximately 150 days after infection or vaccination (Figure 4). Conversely, infection + vaccination participants exhibited a relatively slower rate of linear decline in IgA levels that started to slow around 250 days. Only the infection + vaccination group displayed anti-RBD IgA levels that remained higher than the infection-only group when marginalized over the course of follow-up; on average, levels were 2.6 (95% CI: 2.1–3.1) times higher (Table S3). IgA in vaccination-only participants declined below infection-only participants by 6 months post-vaccination (*p* = 0.007) (Figure 3C). RBD-specific IgA levels were highest in the infection + vaccination group at every time point out to 6 months (all *p* < 0.001) but were indistinguishable from the infection-only group by 9 months post-vaccination (*p* = 0.10).

### 3.4. Serum Neutralizing Activity Is More Durable in Vaccinated Individuals with Prior Natural Immunity out to 9 Months Post-Vaccination

We evaluated neutralization activity in longitudinal samples from participants who provided serum through 9 months post-vaccination or COVID-19 onset. In total, 22, 31, and 11 individuals from the infection-only, vaccination-only, and infection + vaccination groups, respectively, were analyzed. All infection-only participants were positive for serum neutralizing activity by 3 months post-infection but this proportion dropped to 86.4% by 9 months (Table 2). Positive neutralizing activity was retained in most vaccinated participants over time but 9.7% (3/31) of vaccination-only participants became negative by 9 months.

**Figure 5:**
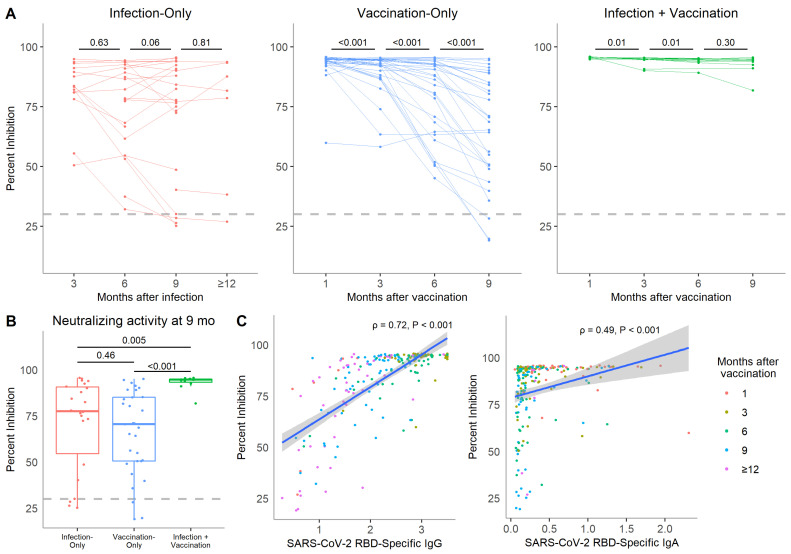
In vitro neutralizing activity in the serum of COVID-19 mRNA vaccinated, SARS-CoV-2 recovered, or both individuals over time. (**A**) SARS-CoV-2 neutralizing activity in paired samples from a subgroup of 22 infection-only participants, 31 vaccination-only participants, and 11 infection + vaccination participants. Neutralizing activity is presented as percent inhibition. The threshold for positive neutralizing activity was set at 30% inhibition based on the manufacturer’s protocol. (**B**) Neutralizing activity at 9 months post-vaccination or infection was compared among the infection-only, vaccination-only, and infection + vaccination groups. (**C**) Spearman correlations between neutralizing activity and RBD-specific IgG and RBD-specific IgA from all 234 serum samples that were analyzed for neutralizing activity are shown. Colors correspond to the time past infection or vaccination. *p*-values were calculated by Wilcoxon signed-rank tests if between paired time points or the Kruskal−Wallis test followed by Dunn’s multiple comparisons test if between multiple groups.

There was no significant difference in neutralization from 3 to 12 months post-COVID-19 onset in the infection-only group (Figure 5A). For vaccination-only participants, neutralizing activity was highest at 1 month but significantly decreased out to 9 months post-vaccination (all *p* < 0.001). Conversely, infection + vaccination participants retained high neutralizing activity at all time points despite a slight decrease from 1 to 6 months post-vaccination (both *p* = 0.01). Neutralization activity at 9 months was highest in the infection + vaccination group and indistinguishable between the infection-only and vaccination-only groups (Figure 5B).

The infection-only group showed the highest variation in neutralizing activity out to 6 months post-vaccination and the infection + vaccination group maintained low variation at all time points (Figure S1). Initially, variation was low in the vaccination-only group but increased over time as neutralizing activity decreased. There was a stronger correlation between neutralization activity and serum levels of RBD-specific IgG than with IgA when looking across all samples (ρ = 0.71 vs. ρ = 0.49, respectively, both *p* < 0.001) (Figure 5C).

## 4. Discussion

This analysis sought to investigate differences in participants’ humoral responses based on their experience with SARS-CoV-2 infection, COVID-19 mRNA vaccination, or both through 9 and 12 months following vaccination and infection, respectively. In the unvaccinated natural infection cohort, we found that SARS-CoV-2 spike-specific IgG levels were highly variable but persisted at modest levels throughout the first year of COVID-19 convalescence. Serum anti-RBD IgA declined in this group from 1 to 3 months but stabilized thereafter. This was reflected in highly variable but detectable neutralizing activity in the serum of most infection-only individuals to a year following infection. Previous studies have also found heterogeneous yet relatively stable SARS-CoV-2-specific antibody responses following infection [5,6,7,35]. In contrast, vaccination induced a predictable immune response in both COVID-19 naïve and experienced individuals. This supports COVID-19 vaccination in SARS-CoV-2 recovered individuals as the strength of an individual’s antibody response following natural infection is unpredictable.

In SARS-CoV-2 naïve vaccine recipients, spike-specific IgG levels continuously declined through at least 9 months post-vaccination where they reached levels seen in infection-only serum at the same time point. Our data extend reports of declining antibody levels following the primary vaccine series past 6 months, which have prompted recommendations for booster doses [12,36,37,38]. Neutralizing activity in the vaccination-only group was predictably strong at early time points following vaccination. While 90% of vaccination-only participants remained above the positive cut-off, the level of neutralizing activity continually decreased following vaccination. In individuals with prior SARS-CoV-2 infection, both IgG and neutralizing antibody responses to vaccination were significantly more robust than the other groups through 9 months, again underscoring the benefit of COVID-19 vaccination after infection.

The systemic IgA response to SARS-CoV-2 infection peaks within the first month after infection and has been followed for more than a year past recovery [7,39]. Follow-up studies on COVID-19 vaccination-induced IgA have been less extensive [26,40]. We found that vaccination alone induced a transient IgA response in the serum that dropped to levels below the positive threshold in most vaccination-only participants by 9 months post-vaccination. However, the reduction of IgA in circulation does not preclude the presence of respiratory tract localized IgA as one study found discordant high mucosal IgA and low serum antibodies in individuals following influenza infection [22].

Vaccination induced a stronger SARS-CoV-2 IgA response in experienced individuals. The long term kinetics of the IgA response for infection + vaccination individuals after 6 months mirrored the infection-only group, suggesting that localized SARS-CoV-2 infection in the respiratory tract may be necessary for sustained SARS-CoV-2-specific IgA production and mucosal immunity [24]. This warrants further investigation into intranasal COVID-19 vaccinations [41] and the subsequent systemic and mucosal IgA responses over time.

The conclusions drawn from this study are subject to limitations. First, although we tried to test for all symptomatic episodes to identify breakthrough infections or reinfections, asymptomatic infection could have been missed during the follow-up period. Second, while recent studies have shown that higher spike-specific IgG and neutralizing antibodies are correlated with reduced risk of symptomatic COVID-19 [3,4], a decline in serum antibody levels may not predict the disappearance of spike-specific memory B cells, which can rapidly activate after antigen re-encounter and have been observed through at least 6 months post-vaccination [42]. Third, additional dilutions in the ELISA assay may have revealed even higher spike-specific IgG titers at the 1-month time point for infection + vaccination individuals. However, we observed adequate dilution curves for all samples, and the infection + vaccination group was already significantly higher than the vaccination-only group at this early time point so our overall conclusions would not change. Finally, SARS-CoV-2 variants of concern that accumulate mutations in the spike protein, such as the Delta and Omicron variants, have been shown to evade antibody-mediated neutralization [43,44]. Our study examines neutralizing activity against the spike protein of the ancestral Wuhan strain only and therefore does not assume neutralization against newer variants.

## 5. Conclusions

Here, we provided a comprehensive picture of SARS-CoV-2 serum antibody dynamics out to 9 months past vaccination and 12 months past infection. This study is one of the first to show enhanced retention of COVID-19 vaccination-induced serum IgA antibodies out to 9 months in SARS-CoV-2 experienced compared with naïve individuals. We also observed more robust and reliable serological responses following COVID-19 vaccination as compared with the high variability of SARS-CoV-2 infection-elicited antibody responses. Thus, our data support COVID-19 vaccination in SARS-CoV-2 naïve and recovered individuals to promote strong, lasting antibody protection against subsequent infection.

## Figures and Tables

**Table 1:** Participant demographics and clinical characteristics.

	Infection-Only (*n* = 218)	Vaccination-Only (*n* = 143)	Infection + Vaccination (*n* = 122)	*p*-Value ^a^
**Sex**				0.92
Male	83 (38.1)	57 (39.9)	46 (37.7)	
Female	135 (61.9)	86 (60.1)	76 (62.3)	
**Age at Vaccination or COVID-19 Onset**				0.04
12–17	39 (17.9)	42 (29.4)	20 (16.4)	
18–29	64 (29.4)	35 (24.5)	40 (32.8)	
30–54	101 (46.3)	52 (36.4)	56 (45.9)	
≥55	14 (6.4)	14 (9.8)	6 (4.9)	
**Race**				<0.001
Asian	7 (3.2)	28 (19.6)	6 (4.9)	
Black	3 (1.4)	3 (2.1)	1 (0.8)	
White	206 (94.5)	104 (72.7)	111 (91.0)	
Multiple	2 (0.9)	8 (5.6)	4 (3.3)	
**Ethnicity**				<0.001
Hispanic/Latinx	201 (92.2)	64 (44.8)	98 (80.3)	
Non-Hispanic/Latinx	17 (7.8)	79 (55.2)	24 (19.7)	
**Comorbid Condition ^b^**	**54 (24.8)**	**73 (51.0)**	**48 (39.3)**	<0.001
Asthma/Pulmonary	24 (11.0)	22 (15.4)	23 (18.9)	
Cancer	2 (0.9)	7 (4.9)	2 (1.6)	
Cardiovascular	19 (8.7)	10 (7.0)	12 (9.8)	
Diabetes/Other endocrine	21 (9.6)	19 (13.3)	14 (11.5)	
Immunosuppression or autoimmunity	3 (1.4)	16 (11.2)	3 (2.4)	
Other chronic condition	1 (0.5)	7 (4.9)	5 (4.1)	
Pregnancy	4 (1.8)	6 (4.2)	3 (2.5)	
**COVID-19 Vaccine Type (Manufacturer)**				0.18
BNT162b2 (Pfizer-BioNTech)	-	125 (87.4)	99 (81.1)	
mRNA-1273 (Moderna)	-	18 (12.6)	23 (18.9)	

^a^ Chi-squared test. ^b^ At present or in the past.

**Table 2:** Proportions of participants with positive antibody responses or neutralizing activity over total tested at each time point past infection or vaccination.

	Infection-Only % (Positive/Total)	Vaccination-Only % (Positive/Total)	Infection + Vaccination % (Positive/Total)
**RBD-specific IgG ^a^**			
1 month (<60 days)	98.8 (81/82)	99.3 (138/139)	100 (78/78)
3 months (60–149 days)	99.3 (140/141)	99.2 (121/122)	100 (86/86)
6 months (150–239 days)	98.7 (74/75)	100 (79/79)	100 (50/50)
9 months (240–329 days)	100 (35/35)	100 (35/35)	100 (12/12)
12 months (≥330 days)	100 (10/10)	-	-
**RBD-specific IgA ^a^**			
1 month (<60 days)	92.7 (76/82)	95.0 (132/139)	100 (78/78)
3 months (60–149 days)	79.4 (112/141)	83.6 (102/122)	98.8 (85/86)
6 months (150–239 days)	85.3 (64/75)	60.8 (48/79)	98.0 (49/50)
9 months (240–329 days)	88.6 (31/35)	37.1 (13/35)	91.7 (11/12)
12 months (≥330 days)	100 (10/10)	-	-
**Neutralizing activity ^b^**			
1 month (<60 days)	100 (3/3)	100 (31/31)	100 (10/10)
3 months (60–149 days)	100 (15/15)	100 (31/31)	100 (11/11)
6 months (150–239 days)	100 (21/21)	100 (30/30)	100 (10/10)
9 months (240–329 days)	86.4 (19/22)	90.3 (28/31)	100 (11/11)
12 months (≥330 days)	85.7 (6/7)	-	-

^a^ Positive thresholds set at 0.200 OD_490_ for RBD-specific IgG and 0.104 OD_490_ for RBD-specific IgA based on mean plus 3 standard deviations from 20 pre-pandemic serum samples. ^b^ Neutralization assay only performed in samples from individuals who provided blood through the 9-month time point. Threshold for positive neutralizing activity set at 30% inhibition based on manufacturer’s protocol.

## Data Availability

The data in this study is available on request from the corresponding author.

## Supplementary Materials

The following supporting information can be downloaded at: https://www.mdpi.com/article/10.3390/vaccines10050813/s1, Table S1: Time intervals for serum collection after COVID-19 onset or vaccination. Table S2: Time between positive COVID-19 test and first vaccine dose for individuals in the infection + vaccination group. Table S3: Characteristics predicting higher levels of serum SARS-CoV-2-specific antibodies. Figure S1: Change in the variation of SARS-CoV-2-specific antibody responses over time following infection or vaccination or both. Figure S2: Correlation between serum SARS-CoV-2 IgG and IgA levels.
