# Supplementary material for: Twelve-Month Longitudinal Serology in SARS-CoV-2 Naïve and Experienced Vaccine Recipients and Unvaccinated COVID-19-Infected Individuals
Source: Vaccines (Basel). 2022 May 20;10(5):813. doi: 10.3390/vaccines10050813 (PMC9143304; doi:10.3390/vaccines10050813)
Supplement: Supplementary file 1 [file vaccines-10-00813-s001.zip › vaccines-1722654-supplementary.pdf]

Supplemental Tables

Table S1. Time intervals for serum collection after COVID-19 onset or vaccination.

**Time passed since infection or vaccination**	**Infection-Only** (n=343)		**Vaccination-Only** (n=375)		**Infection + Vaccination** (n=226)	
	n	Median days (IQR)	n	Median days (IQR)	n	Median days (IQR)
1 month (<60 days)	82	22 (16-46)	139	36 (34-42)	78	36 (34-40)
3 months (60 – 149 days)	141	87 (74-95)	122	94 (91-101)	86	95 (90-117)
6 months (150 – 239 days)	75	174 (165-194)	79	184 (182-192)	50	189 (181-206)
9 months (240 – 329 days)	35	281 (265-307)	35	273 (266-274)	12	267 (260-275)
12 months (≥330 days)	10	390 (363-413)	-	-	-	-

Table S2. Time between positive COVID-19 test and first vaccine dose for individuals in the infection + vaccination group.

**Time between positive COVID-19 test and first vaccine dose**	**n (%)**
0-3 months	19 (15.6)
3-6 months	23 (18.9)
6-9 months	28 (23.0)
9-12 months	17 (13.9)
12+ months	6 (4.9)
Unknown	29 (23.8)

Table S3. Characteristics predicting higher levels of serum SARS-CoV-2-specific antibodies.

	Spike-specific IgG		RBD-specific IgA	
**Characteristic**	**Odds ratio**	**95% CI**	**Odds ratio**	**95% CI**
**Age** (years)				
12-17	1 (Ref)	-	1 (Ref)	-
18-29	0.56	0.39 - 0.81	0.86	0.68-1.09
30-54	0.68	0.48 - 0.95	0.92	0.74-1.15
≥55	0.71	0.42 - 1.19	1.02	0.73-1.44
**Sex**				
Male	1 (Ref)	-	1 (Ref)	-
Female	0.86	0.66 - 1.14	0.68	0.57-0.81
**Infection/vaccination status**				
Infection-Only	1 (Ref)	-	1 (Ref)	-
Vaccination-Only	6.38	4.74 - 8.60	1.03	0.85-1.25
Infection + Vaccination	15.19	10.90 - 21.16	2.55	2.07-3.13

Supplemental Figures

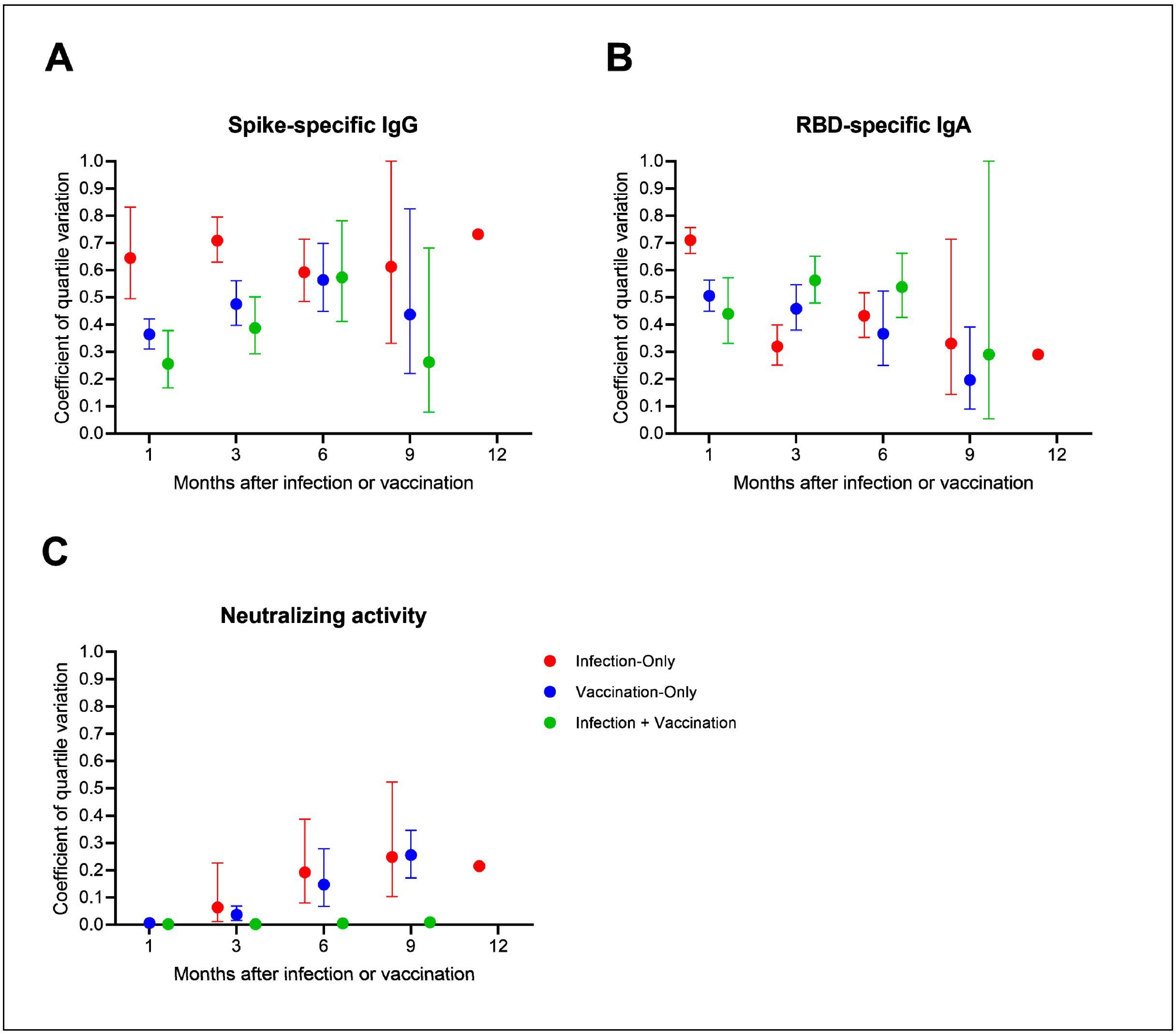

Figure S1. Change in the variation of SARS-CoV-2-specific antibody responses over time following infection or vaccination or both. Coefficients of quartile variation (CQVs) for **(A)** spike-specific IgG, **(B)** RBD-specific IgA, and **(C)** serum neutralizing activity at each time point past vaccination or infection are presented for participants in the infection-only, vaccination-only, and infection + vaccination groups. Dots are the CQV, calculated using the interquartile range for each group at each time point. Error bars represent 95% confidence intervals determined by Bonnett's method. Color corresponds with group.

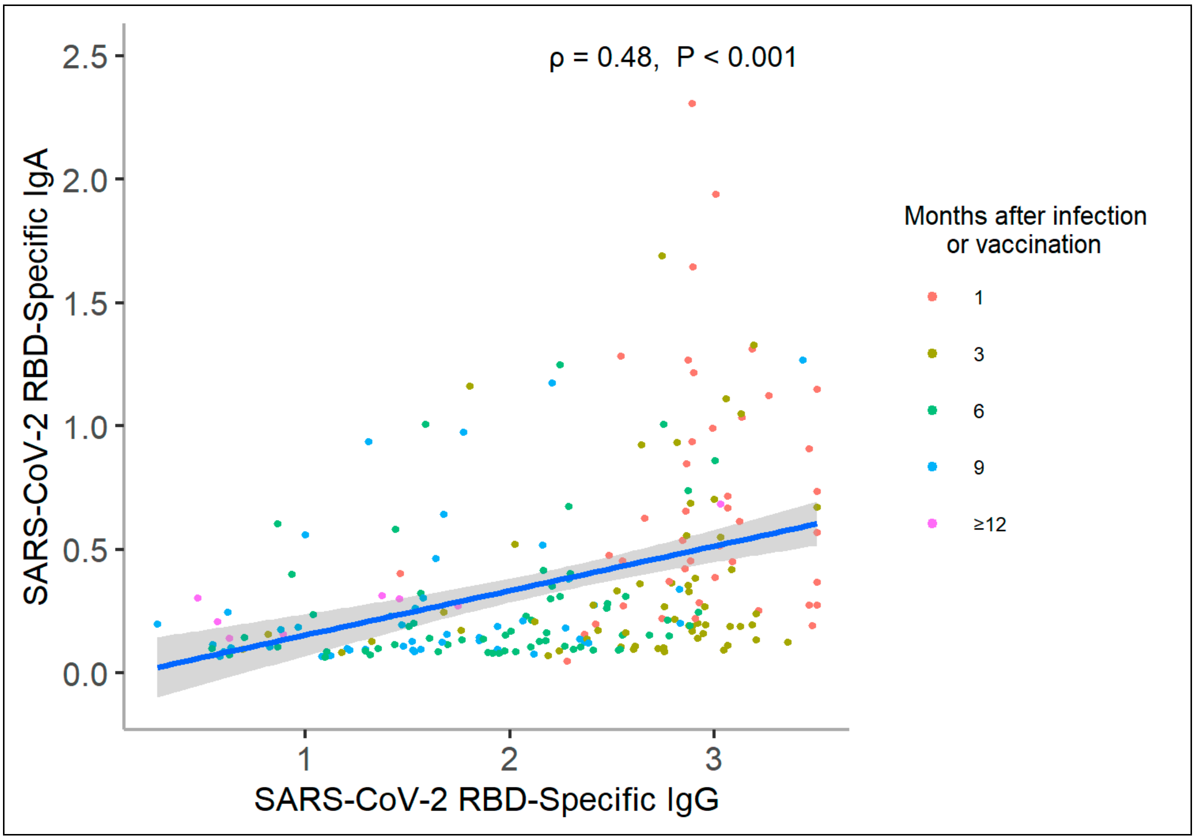

Figure S2. Correlation between serum SARS-CoV-2 IgG and IgA levels. Correlation between SARS-CoV-2-specific IgG and IgA in the same serum sample was determined using Spearman's correlation coefficient. Color corresponds to month past infection or vaccination.
